# Giant Intra-Abdominal Desmoid Tumor in a Young Man: A Case Report and Literature Review

**DOI:** 10.70352/scrj.cr.24-0019

**Published:** 2025-03-15

**Authors:** Yusuke Tanaka, Takahiro Toyokawa, Mami Yoshii, Yuichiro Miki, Tatsuro Tamura, Shigeru Lee, Kiyoshi Maeda

**Affiliations:** 1 Department of Gastroenterological Surgery, Tsukazaki Hospital, Himeji, Hyogo, Japan; 2 Department of Gastroenterological Surgery, Osaka Metropolitan University Graduate School of Medicine, Osaka, Osaka, Japan

**Keywords:** Desmoid tumor, giant, sporadic, fibromatosis

## Abstract

**INTRODUCTION:**

Desmoid tumors are rare soft-tissue tumors with a high recurrence rate; however, histologically, these tumors are benign. We describe a case in which a giant desmoid tumor was resected in a young man without any apparent causative factors.

**CASE PRESENTATION:**

A 21-year-old man was referred to our hospital for treatment after presenting to a nearby hospital with right inguinal pain. Abdominal magnetic resonance imaging showed an intra-abdominal mass measuring 34 × 15 × 8 cm with partial signal hyperintensity on T2-weighted imaging and hypointensity on T1-weighted imaging, extending from the left abdominal cavity to the pelvic region. Although no definitive diagnosis was obtained preoperatively, surgery was performed under suspicion of gastrointestinal stromal tumor or other significant disease. A mass was identified firmly adherent to the transverse colon, gastric wall, and diaphragm, and these organs were partially resected. The excised specimen measured 38 × 21 × 8 cm and weighed 6400 g. Macroscopically, the tumor showed a smooth surface and homogeneous interior. Pathological examination revealed atypical cells with spindle-shaped nuclei and collagen fiber hyperplasia in the stroma, and immunostaining was negative for c-kit, CD34, desmin, S-100, and positive for β-catenin, leading to a confirmed diagnosis of desmoid tumor. Fifteen months after surgery, a local recurrence with a diameter of 3.0 cm was identified, and the patient remains under careful follow-up.

**CONCLUSIONS:**

Intra-abdominal desmoid tumors larger than 30 cm are extremely rare. When encountering a young patient with a large intra-abdominal tumor, the possibility of desmoid tumor should be considered.

## Abbreviations

CT
: computed tomography
EUS-FNA
: endoscopic ultrasound-guided fine-needle aspiration
FAP
: familial adenomatous polyposis
MRI
: magnetic resonance imaging
NCCN
: National Comprehensive Cancer Network
NSAID
: non-steroidal anti-inflammatory drug

## INTRODUCTION

Desmoid tumors are stromal tumors arising from fascial and myotendinous tissue and were first described by McFarlane in 1833.^1)^ Desmoid tumors are histologically benign but are known as aggressive fibromatosis that tends to infiltrate locally, leading to a high local recurrence rate even after complete resection, but they have no metastatic potential.^2)^ Desmoid tumors are rare, with an incidence of 2.4–4.3 cases per million persons per year. This pathology accounts for approximately 0.03% of all neoplasms and less than 3% of all soft-tissue tumors.^3)^ Although specific pathogenic mechanisms have not been clarified, familial adenomatous polyposis (FAP) and Gardner syndrome are widely known to be associated with the development of hereditary desmoid tumors, along with histories of laparotomy and trauma, while pregnancy is associated with the development of sporadic cases.^4)^ According to their location, desmoid tumors are clinically categorized as extra-abdominal, superficial (abdominal wall), or intra-abdominal.^5)^ Intra-abdominal desmoid tumors are the rarest of these and have the poorest prognosis, making the management of this disease is challenging.

We report herein an extremely rare case involving a 21-year-old man with a giant intra-abdominal desmoid tumor more than 30 cm in diameter without any apparent causative factors.

## CASE PRESENTATION

**Fig. 1:**
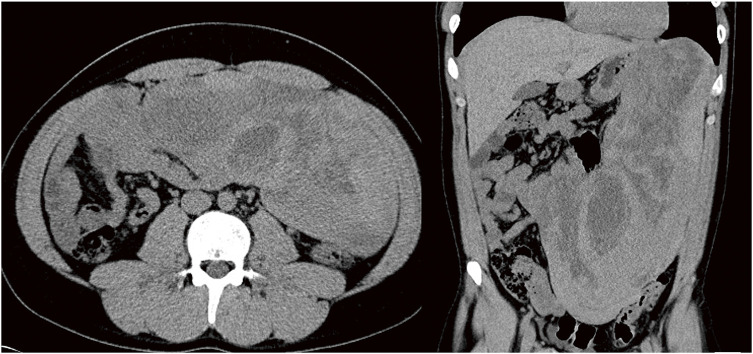
Abdominal CT shows a well-defined mass up to 34 cm in diameter, extending from the left diaphragm to the pelvic cavity. The interior of the mass shows speckled absorption values and no calcification. CT, computed tomography

**Fig. 2:**
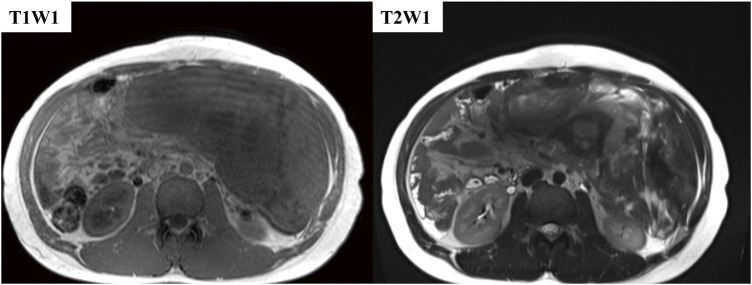
Abdominal MRI shows an intra-abdominal tumor measuring 34 × 15 × 8 cm with a low signal on T2W1, partially hyperintensity on T1W1, and isointensity on T1W1. No internal hemorrhage or fatty components are apparent. MRI, magnetic resonance imaging; T1W1, T1-weighted imaging; T2W1, T2-weighted imaging

A 21-year-old man visited a clinic with a chief complaint of right inguinal pain. Abdominal ultrasonography revealed a huge intra-abdominal mass, and he was referred to our hospital for further examination. He had no specific family history but had a history of autism spectrum disorder and bronchial asthma, with no history of trauma or abdominal surgery. The abdomen was mildly distended and soft, and the tumor was not palpable. Laboratory examinations showed no abnormalities in blood count, biochemistry, or coagulation markers, no elevation of tumor markers, and no elevation of immunoglobulins. Abdominopelvic computed tomography (CT) showed a huge mass with a maximum diameter of 34 cm and well-defined borders, extending from the upper abdomen to the pelvis (**Fig. 1**). Magnetic resonance imaging (MRI) revealed an intra-abdominal tumor 34 × 15 × 8 cm with partial signal hyperintensity on T2-weighted imaging, hypointensity on T1-weighted imaging (**Fig. 2**). No internal calcification, fatty, or blood components were evident, and a portion of the tumor appeared to be in contact with the gastric body. Due to the history of bronchial asthma, a contrast-enhanced examination could not be performed. Endoscopic ultrasonography showed a heterogeneous hypoechoic internal cavity, but endoscopic ultrasound-guided fine-needle aspiration (EUS-FNA) was not performed, given the risk of seeding. Although no definitive diagnosis or organ of origin was confirmed preoperatively for the tumor, we performed surgery under suspicion of gastrointestinal stromal tumor, desmoid tumor, or mesenteric tumor.

**Fig. 3:**
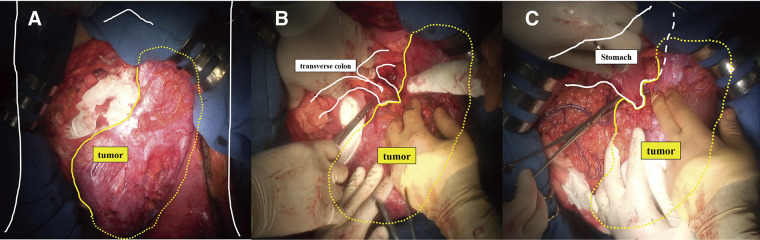
(**A)** Preoperative imaging shows the tumor occupying much of the abdominal cavity. (**B)** Adhesions are observed slightly on the left side of the transverse colon, and partial resection of the transverse colon is performed. (**C)** Adhesions are observed on the greater curvature of the gastric body, and the stomach is partially resected.

**Fig. 4:**
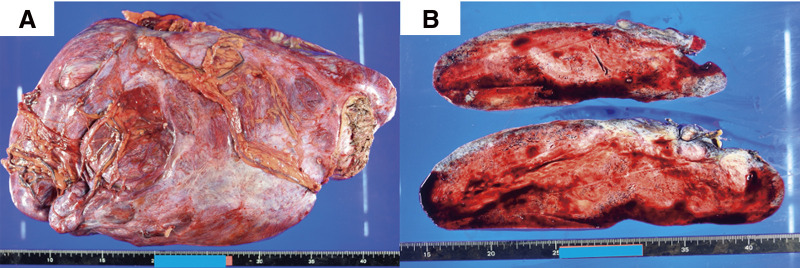
(**A)** The excised tumor is 38 × 21 × 8 cm in size and weighs 6400 g. (**B)** The surface is smooth and the interior is homogeneously filled with tumors.

**Fig. 5:**
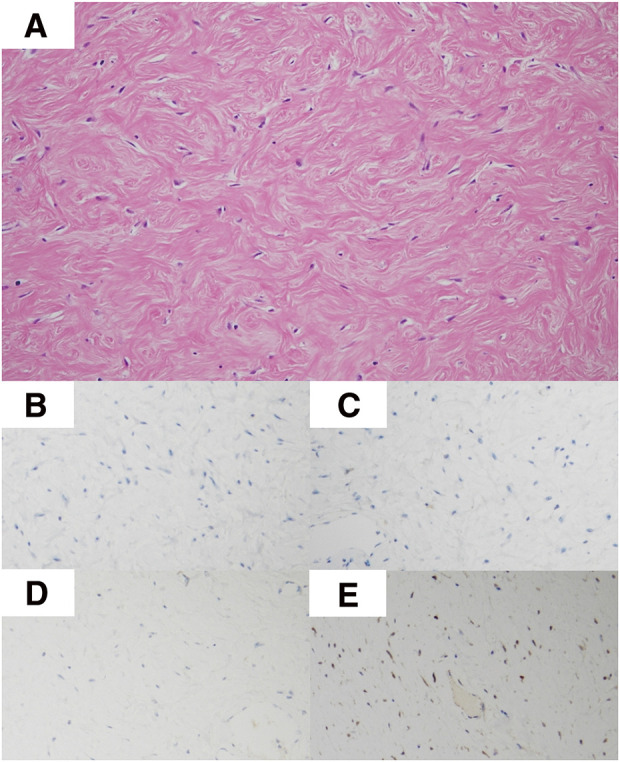
(**A)** Hematoxylin–eosin staining reveals a conspicuous increase in collagen and a coarse observation of dysmorphic cells with oval to spindle-like nuclei (magnification ×10). Immunostaining for (**B**) CD34, (**C**) desmin, (**D**) S-100, and (**E)** β-catenin shows negative results (magnification ×10).

The patient was placed in the supine position, and laparotomy was performed through upper and lower median incisions and a left transverse incision. Intraoperative findings showed a giant tumor covered with a capsule occupying most of the left side of the abdominal cavity, extending from the pelvis to the diaphragm. The tumor showed firm adhesions to the stomach, transverse colon, and diaphragm without invasion of other organs. The omentum, with dilated vessels, was also adherent to the tumor, so the tumor had to be partially resected en bloc with portions of the stomach, transverse colon, and diaphragm (**Fig. 3**). After tumor resection, primary anastomosis of the colon and closure of the diaphragm were performed. The tumor appeared to have been completely excised without rupture of the capsule. The operative time was 3 h 57 min, with a total blood loss of 110 mL. The excised specimen measured 38 × 21 × 8 cm and weighed 6400 g. The surface was smooth, and the interior was homogeneously filled with tumor on macroscopic examination (**Fig. 4**). Pathological examination revealed atypical cells with spindle-shaped nuclei and collagen fiber hyperplasia in the stroma. Immunostaining showed negative results for c-kit, CD34, desmin, and S-100, while positive results for β-catenin confirmed the diagnosis of a desmoid tumor (**Fig. 5**). Pathological results for the surgical margins were negative, confirming complete surgical resection. Despite pathological examination of the resected organs, determining the primary site of tumor origin was difficult. The patient was discharged 17 days after surgery, despite the appearance of surgical site infection. Colonoscopy revealed no polyps. A CT scan conducted 12 months after surgery showed a nodule 3.0 cm in diameter near the suture line of the gastric body; however, its size remained unchanged on a subsequent CT scan taken 9 months later, 21 months postoperatively. The patient is under close follow-up.

## DISCUSSION

Desmoid tumors are defined as: i) proliferation of highly differentiated fibroblasts; ii) absence of cytologic evidence of malignancy or marked nuclear fission; iii) presence of large amounts of collagen fibers among proliferating cells; iv) an infiltrative growth pattern; and v) absence of metastasis but a high likelihood of local recurrence.^6)^ Based on classifications established by the World Health Organization, a desmoid tumor is classified as a soft-tissue tumor intermediate between benign and malignant. The tumor is most common in those between 15 and 60 years old, rare in the young and elderly, and slightly more common in women, with no significant racial or ethnic predilections identified.^7)^

The etiology of desmoid tumors is unknown; however, they are known to be associated with FAP, mechanical stimuli such as open surgery or trauma, estrogenic tumors, and increased estrogen levels related to pregnancy.^4)^ In patients with FAP, germline mutations in the *APC* gene are involved in the development of desmoid tumors, while sporadic intra-abdominal desmoid tumors are attributed to somatic mutations in *CTNNB1*. These mutations were reportedly observed in 77.3% of desmoid tumor cases in a large French cohort study.^8)^ With both mutations, the degradation of β-catenin is inhibited, and the stabilization of β-catenin is believed to result in the formation of desmoid tumors. Approximately 10% of patients with FAP develop desmoid tumors,^9)^ and the risk of developing a desmoid tumor is 852 times greater in patients with FAP than in the general population.^10)^ On the other hand, the probability of detecting FAP in patients who developed desmoid tumor without a history of FAP is reportedly relatively low, at 3.7%.^11)^ Therefore, aggressive colonoscopy is unnecessary in non-FAP patients with desmoid tumors. This case did not involve FAP-related desmoid tumors.

We did not perform EUS-FNA or needle biopsy in the present case because of concerns of tumor cell seeding; however, no reports have identified specific associations between needle biopsy and tumor seeding for desmoid tumors. The only study we could identify regarding gastrointestinal stromal tumors demonstrated that percutaneous needle biopsy was not associated with an increased risk of relapse-free survival.^12)^ On the other hand, van Houdt et al. reported that the risk of needle tract seeding after trans-abdominal needle biopsy for retroperitoneal sarcoma was low but not zero, at 2%.^13)^ The necessity of EUS-FNA and needle biopsy to confirm the preoperative diagnosis is debatable in cases like the present one, as the removal of such a large tumor associated with symptoms would be desirable even for a benign tumor.

Complete surgical resection has been widely accepted as the first-line treatment for desmoid tumors. However, solid evidence that surgery offers superior prognosis over other modalities has not yet been established. The local recurrence rate after surgery for desmoid tumors is reportedly 30%–40%.^3)^ As in our case, young age and large tumor size have been reported as risk factors for local recurrence.^14,15)^ Regarding the relationship between the state of the resected margin and local recurrence, Salas et al. reported 2- and 5-year progression-free survival rates of 77% and 63% in patients with R0 resection, which were not significantly different from the rates of 74% and 63% in patients with R1 resection. In contrast, rates of 43% and 22% were significantly lower in patients with R2 resection in a large series of 426 newly diagnosed desmoid tumors.^15)^ Surgery represents a type of trauma, which could be a causative factor for desmoid tumor and may contribute to a high local recurrence rate. On the other hand, the clinical course of desmoid tumor has often revealed spontaneous regression or long-lasting stable disease,^16,17)^ and recent reports have clarified the efficacy of a wait-and-see approach for desmoid tumors.^8,18)^ Nowadays, major treatment guidelines from the National Comprehensive Cancer Network (NCCN) and the Desmoid Tumor Working Group both recommend an observational approach as the preferred front-line management for asymptomatic, non-progressing desmoid tumors.^2)^ In the present case, although the chief complaint of the patient was mild, we decided on up-front surgery because a definitive diagnosis was difficult to reach and the risk of surgery was considered likely to increase if the tumor grew any larger.

Regarding the follow-up interval and periods, the NCCN guidelines recommend follow-up with CT or MRI every 3–6 months for the first 2–3 years after surgical resection of a desmoid tumor and every 6–12 months thereafter.

The optimal treatment strategy for recurrent desmoid tumors has not been established. Tsagozis et al. revealed that surgery for recurrent desmoid tumors carries a markedly high risk of local recurrence (93%), irrespective of the surgical margin, and recommended observation or medical treatment for asymptomatic patients at high risk of surgical morbidities, as stable disease is common and spontaneous regression can be expected in a small number of patients.^19)^ Medical treatments include non-steroidal anti-inflammatory drugs (NSAIDs), hormonal agents, cytotoxic chemotherapy, and tyrosine kinase inhibitors. Retrospective studies have demonstrated the efficacy of NSAIDs and hormonal agents, but prospective evaluations of their efficacy compared to the wait-and-see approach remain lacking.^3)^ Conventional chemotherapy and tyrosine kinase inhibitors are used for patients with rapidly growing and symptomatic but unresectable tumors.^2)^ In the present case, we suggested careful follow-up observation because the recurrent nodule was small and located in an area considered easily amenable to resection. As of the time of writing, 9 months after identification, the recurrent nodule has shown no change in size. We intend to perform radical surgical treatment if the nodule increases in size, as we believe that the recurrent tumor can be readily removed without impairing quality of life or organ function due to its location.

A literature search using the PubMed database found only 8 case reports (including the present report) describing intra-abdominal giant desmoid tumors larger than 30 cm in diameter^20–26)^ (**Table 1**). The median age in these 8 cases was 24.5 years (range, 14–38 years), indicating that as-yet-unidentified genetic factors may be involved in the development of giant intra-abdominal desmoid tumors. These 8 cases occurred in 3 female patients and 5 male patients. One of the 3 female patients had Gardner syndrome and had been pregnant, while another was pregnant. Intra-abdominal desmoid tumors without causative factors appear more frequent in males. All patients were symptomatic, with pain being the most common complaint due to tumor size. Preoperative biopsies were performed in only 3 cases, and only 1 case was diagnosed preoperatively as desmoid. Most cases needed bowel resection. Although most cases originated from the mesentery, we were unable to identify the primary site of origin in the present case. Among the 8 cases, recurrence was observed in only our case during the median postoperative observation period of 13.5 months (range, 5–54 months). Although we were unable to identify the primary site of the tumor from the surgical findings and pathological results, the recurrence near the suture line of the gastric body may indicate that the stomach was the primary site.

**Table 1:** Seven cases of intra-abdominal giant desmoid tumors larger than 30 cm published in the English literature

Author	Year	Age	Gender	Symptom	Preoperative biopsy	Size (resected specimen)	Primary site	Postoperative observational period	Trigger	Operation
de Bree et al.^20)^	2013	31	Female	Abdominal distension	Myofibroblastic lesion	33 cm	Transversal mesocolon	54 months	Pregnant	Tumor resection
Williams et al.^21)^	2016	33	Male	Abdominal pain	Neoplastic spindle cells	45 cm	Iliocolic mesentery	Not available	Sporadic	Omentectomy Ileocecectomy
Mizuta and Tsunemi^22)^	2018	17	Male	Abdominal pain	None	30 cm	Transversal mesocolon	Not available	Sporadic	Partial gastrectomy Pancreas caudal resection
Jin et al.^23)^	2020	28	Female	Upper abdominal pain	None	30 cm	Unknown	6 months	Gardner Syn Pregnant	Tumor resection
Elhaddad et al.^24)^	2022	38	Male	Abdominal pain, dyspnea on exertion	None	40 cm	Iliocolic mesentery	5 months	Sporadic	Right partial colectomy
Rabai et al.^25)^	2023	14	Female	Abdominal pain	None	30 cm	Iliocolic mesentery	12 months	Sporadic	Partial resection of the small bowel
Kim et al.^26)^	2023	18	Male	Upper abdominal discomfort	Desmoid-type fibromatosis	38 cm	Iliocolic mesentery	30 months	Sporadic	Ileocecectomy
Our case	2023	21	Male	Inguinal pain	None	38 cm	Unknown	18 months	—	Tumor resection with partial resection of the stomach, transverse colon, and diaphragm

## CONCLUSIONS

We encountered a case involving a sporadic giant intra-abdominal desmoid tumor exceeding 30 cm in diameter. When a young patient presents with a large intra-abdominal tumor, the possibility of desmoid tumor must be considered. Currently, surgery is not always the first choice of treatment for a desmoid tumor, but it should be considered when the tumor adversely affects the patient’s quality of life and the risk of surgery is likely to increase with further tumor growth. This appears to be the only report to identify recurrence after surgery for an intra-abdominal desmoid tumor larger than 30 cm, and we plan to follow the patient for a long period.

## ACKNOWLEDGMENTS

The authors acknowledge all the ward staff who took care of the patient. We also thank Dr. Kaori Sakamoto for the histopathological diagnosis.

## DECLARATIONS

### Funding

Funding information is not applicable.

### Authors’ contributions

YT and TT1 drafted the manuscript.

MY, YM, TT2, and SL participated in its design and coordination and helped to draft the manuscript.

KM contributed to critical revision.

All authors read and approved the final manuscript.

All authors agree to be responsible for all aspects of the study.

### Availability of data and materials

Not applicable.

### Ethics approval and consent to participate

The study procedures adhered to the tenets of the Declaration of Helsinki.

### Consent for publication

Written informed consent was obtained from the patient for publication of this case report and any accompanying images.

### Competing interests

The authors declare that they have no competing interests.
